# Auto-validating von Neumann rejection sampling from small phylogenetic tree spaces

**DOI:** 10.1186/1748-7188-4-1

**Published:** 2009-01-07

**Authors:** Raazesh Sainudiin, Thomas York

**Affiliations:** 1 Department of Statistics, University of Oxford, Oxford, OX1 3TG, UK; 2 Biomathematics Research Centre, Department of Mathematics and Statistics, University of Canterbury, Private Bag 4800, Christchurch, New Zealand; 3 Department of Biological Statistics and Computational Biology, Cornell University, Ithaca, New York 14853, USA; 4 Boyce Thompson Institute for Plant Research, Cornell University, Ithaca, New York 14853, USA

## Abstract

**Background:**

In phylogenetic inference one is interested in obtaining samples from the posterior distribution over the tree space on the basis of some observed DNA sequence data. One of the simplest sampling methods is the rejection sampler due to von Neumann. Here we introduce an auto-validating version of the rejection sampler, via interval analysis, to rigorously draw samples from posterior distributions over small phylogenetic tree spaces.

**Results:**

The posterior samples from the auto-validating sampler are used to rigorously (i) estimate posterior probabilities for different rooted topologies based on mitochondrial DNA from human, chimpanzee and gorilla, (ii) conduct a non-parametric test of rate variation between protein-coding and tRNA-coding sites from three primates and (iii) obtain a posterior estimate of the human-neanderthal divergence time.

**Conclusion:**

This solves the open problem of rigorously drawing independent and identically distributed samples from the posterior distribution over rooted and unrooted small tree spaces (3 or 4 taxa) based on any multiply-aligned sequence data.

## Background

Obtaining samples from a real-valued target density *f*^•^ (*t*) is a basic problem in statistical estimation. The target *f*^• ^(*t*): T ↦ ℝ maps *n*-dimensional real points in ℝ^*n *^to real numbers in ℝ, i.e. *t *∈ T ⊂ ℝ^*n*^. In Bayesian phylogenetic estimation, we want to draw independent and identically distributed samples from a target posterior density on the space of phylogenetic trees. The standard point-valued or punctual Monte Carlo methods via conventional floating-point arithmetic are typically non-rigorous as they do not account for all sources of numerical errors and are limited to evaluating the target at finitely many points. The standard approaches to sampling from the posterior density, especially over phylogenetic trees, rely on Markov chain Monte Carlo (MCMC) methods. Despite their asymptotic validity, it is nontrivial to guarantee that an MCMC algorithm has converged to stationarity [1], and thus MCMC convergence diagnostics on phylogenetic tree spaces are heuristic [2].

A more direct sampler that is capable of producing independent and identically distributed samples from the target density *f*^• ^(*t*):= *f*(*t*)/(*N*_*f*_), by only evaluating the target shape *f*(*t*) without knowing the normalizing constant $N_{f}:=\int_{T}f(t)dt$, is the von Neumann rejection sampler [3]. However, the limiting step in the rejection sampler is the construction of an envelope function $\hat{g}$(*t*) that is not only greater than the target shape *f*(*t*):= *N*_*f *_*f*^• ^(*t*) at every *t *∈ T, but also easy to normalize and draw samples from. Moreover, a practical and efficient envelope function has to be as close to the target shape as possible from above. When an envelope function is constructed using point-valued methods, except for simple classes of targets, one cannot guarantee that the envelope function dominates the target shape globally.

None of the available samplers can rigorously produce independent and identically distributed samples from the posterior distribution over phylogenetic tree spaces, even for 3 or 4 taxa. We describe a new approach for rigorously drawing samples from a target posterior distribution over small phylogenetic tree spaces using the theory of *interval analysis*. This method can circumvent the problems associated with (i) heuristic convergence diagnostics in MCMC samplers and (ii) pseudo-envelopes constructed via non-rigorous point-valued methods in rejection samplers.

Informally, our method partitions the domain into boxes and uses interval analysis to rigorously bound the target shape in each box; then we use as envelope the simple function which takes on in each box the upper bound obtained for that box. It is easy to draw samples from the density corresponding to this step function envelope. More formally, the method employs an interval extension of the target posterior shape *f*(*t*): T ↦ ℝ to produce rigorous enclosures of the range of *f *over each interval vector or box in an adaptive partition $T:=\{t^{\left(1\right)},t^{\left(2\right)},...,t^{\left(|T|\right)}\}$ of the tree space T = ∪_*i *_***t***^(*i*)^. This partition is adaptively constructed by a priority queue. The interval extended target shape maps boxes in T to intervals in ℝ. This image interval provides an upper bound for the global maximum and a lower bound for the global minimum of *f *over each element of the partition of T. We use this information to construct an envelope as a simple function over the partition T. Using the Alias method [4] we efficiently propose samples from this normalized step-function envelope for von Neumann rejection sampling.

We call our method auto-validating because we employ interval methods to rigorously construct the envelope for a large class of target densities. The method was described in a more rudimentary form in [5]. Unlike many conventional samplers, each sample produced by our method is equivalent to a computer-assisted proof that it is drawn from the desired target, up to the pseudo-randomness of the underlying, deterministic, pseudo-random number generator. MRS 0.1.2, a C++ class library for statistical set processing is available from http://www.math.canterbury.ac.nz/~r.sainudiin/codes/mrs under the terms of the GNU General Public License.

The rest of the paper is organized as follows. In the Methods Section, we introduce (i) von Neumann rejection sampler (RS), (ii) phylogenetic estimation problem, (iii) interval analysis and (iv) an interval extension of the rejection sampler called the Moore rejection sampler (MRS) in honor of Ramon E. Moore. Moore was one of the influential founders of interval analysis [6]. In Results Section, we employ MRS to rigorously draw samples from the posterior density over small tree spaces. Using one of the earliest primate mitochondrial DNA data sets we use the posterior samples to estimate the posterior probability of each rooted tree topology and conduct a non-parametric test of rate variation between protein-coding and tRNA-coding sites. Using one of the latest data sets we obtain a rigorous posterior estimate of the human-neanderthal divergence time. We can also draw samples from the space of unrooted triplet and quartet trees. We conclude after a discussion of the method.

## Methods

In the following sections, we first introduce the rejection sampler (RS) due to von Neumann [3]. Secondly, we describe the basic phylogenetic inference problem (e.g. [7-9]). Then, we introduce the basic principles of interval methods (e.g. [6,10-13]). Finally, we construct interval extensions of RS to rigorously draw independent and identically distributed samples from small phylogenetic tree spaces. We leave the formal proofs to the Appendix for completeness.

### Rejection sampler (RS)

Rejection sampling [3] is a Monte Carlo method to draw independent samples from a target random variable or random vector *T *with density *f*^• ^(*t*):= *f*(*t*)/*N*_*f*_, where *t *∈ T ⊂ ℝ^*n*^, i.e. *T *~ *f*^•^. The challenge is to draw the samples without any knowledge of the normalizing constant $N_{f}:=\int_{T}f(t)dt$. Typically the target *f*^• ^(*t*) is any density that is absolutely continuous with respect to the Lebesgue measure. The von Neumann rejection sampler (RS) can produce samples from *T *~ *f*^• ^according to Algorithm 1 when provided with (i) a fundamental sampler that can produce independent samples from the Uniform [0, 1] random variable *M *with density given by the indicator function **1**_[0,1]_(*m*): ℝ ↦ ℝ, (ii) a target shape *f*(*t*): T ↦ ℝ, (iii) an envelope function $\hat{g}(t):T\mapsto \mathbb{R}$, such that,

(1) $$\begin{array}{ccc} \hat{g}(t)\geq f(t) & \text{for all} & t\in T, \end{array}$$

(iv) a normalizing constant $N_{\hat{g}}:=\int_{T}\hat{g}(t)dt$, (v) a proposal density $g(t):={\left(N_{\hat{g}}\right)}^{-1}\hat{g}(t)$ over T from which independent samples can be drawn and finally (vi) *f*(*t*) and $\hat{g}$(*t*) must be computable for any *t *∈ T.

**input **: (i) *f*; (ii) samplers for *V *~ *g *and *M *~ **1**_[0,1]_; (iii) $\hat{g}$; (iv) integer MaxTrials;

**output **: (i) possibly one sample *t *from *T *~ *f*^• ^and (ii) Trials

**initialize**: Trials ← 0; Success ← false; *t *← ∅;

**repeat **   //propose at most MaxTrials times until acceptance

   *v *← sample(*g*);         //draw a sample *v *from RV *V *with density *g*

   *u *← $\hat{g}$(*v*) sample(**1**_[0,1]_);      //draw a sample *u *from RV *U *with density $1_{\left[0,\hat{g}(v)\right]}$

   **if ***u *≤ *f*(*v*) **then**            //accept the proposed *v *and flag Success

      *t *← *v*; Success ← true

   **end**

   Trials ← Trials +1;         //track the number of proposal trials so far

**until **Trials ≥ MaxTrials *or *Success = true;

**return ***t and *Trials

**Algorithm 1**: von Neumann RS

We use the Mersenne Twister pseudo-random number generator [14] to imitate independent samples from *M *~ **1**_[0,1]_. The random variable *T*, if generated by Algorithm 1, is distributed according to *f*^• ^(e.g. [15]). Let ***A***($\hat{g}$) be the probability that a point proposed according to *g *gets accepted as an independent sample from *f*^• ^through the envelope function $\hat{g}$. Observe that the envelope-specific acceptance probability ***A***($\hat{g}$) is the ratio of the integrals

$$A(\hat{g})=\frac{N_{f}}{N_{\hat{g}}}:=\frac{\int_{T}f(t)dt}{\int_{T}\hat{g}(t)dt},$$

and the probability distribution over the number of samples from *g *to obtain one sample from *f*^• ^is geometrically distributed with mean 1/***A***($\hat{g}$) (e.g. [15]).

### Phylogenetic estimation

In this section we briefly review phylogenetic estimation. A more detailed account can be found in [7-9]. Inferring the ancestral relationship among a set of extant species based on their DNA sequences is a basic problem in phylogenetic estimation. One can obtain the likelihood of a particular phylogenetic tree that relates the extant species of interest at its leaves by superimposing a continuous time Markov chain model of DNA substitution upon that tree. The length of an edge (branch length) connecting two nodes (species) in the tree represents the amount of evolutionary time (divergence) between the two species. The internal nodes represent ancestral species. During the likelihood computation, one needs to integrate over all possible states at the unobserved ancestral nodes.

**Figure 1:**
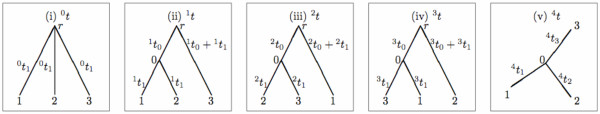
**Tree space with three labeled leaves**. Space of phylogenetic trees with three labeled leaves {1, 2, 3}. See text for description.

Next we give a brief introduction to some phylogenetic nomenclature. A phylogenetic tree is said to be rooted if one of the internal nodes, say node *r*, is identified as the root of the tree, otherwise it is said to be unrooted. The rooted tree is conventionally depicted with the root node *r *at the top. The four topology-labeled, three-leaved, rooted trees, namely, ^0^*t*, ^1^*t*, ^2^*t *and ^3^*t*, with leaf label set {1, 2, 3}, are depicted in Figure 1(i)–(iv). The unrooted, three-leaved tree with topology label 4 or the unrooted triplet ^4^*t *is shown in Figure 1(v). For each tree, the terminal branch lengths, i.e. the branch lengths leading to the leaf nodes, have to be strictly positive and the internal branch lengths have to be non-negative. Our rooted triplets (Figure 1(i)–(iv)) are said to satisfy the molecular clock, since the branch lengths of each ^*k*^*t*, where *k *∈ {0, 1, 2, 3}, satisfy the constraint that the distance from the root node *r *to each of the leaf nodes is equal to ^*k*^*t*_0 _+ ^*k*^*t*_1 _with ^*k*^*t*_1 _> 0 and ^*k*^*t*_0 _≥ 0.

#### Likelihood of a tree

Let *d *denote a homologous set of sequences of length *v *with character set $U=\{a_{1},a_{2},...,a_{\left|U\right|}\}$ from *n *taxa. We think of *d *as an *n *× *v *matrix with entries from U. We are interested in estimating the branch lengths and topologies of the tree underlying our observed *d*. Let *b*_*k *_denote the number of branches and *s*_*k *_denote the number of nodes of a tree with a specific topology or branching order labeled by *k*. Thus, for a given topology label *k*, *n *labeled leaves and *b*_*k *_many branches, the labeled tree ^*k*^*t *is the topology-labeled vector of branch lengths (^*k*^*t*_1_,...,tkbk) contained in the topology-labeled tree space Tk, i.e.,

Tk:={tk:=(tk 1,...,tk bk)∈ℝ+bk: kti>0 for terminal branches}.

Any subset of the tree space with |K| many topologies in the topology label set K can be defined as follows:

TK:=∪k∈KTk.

An explicit model of sequence evolution is prescribed in order to obtain the likelihood of observing data *d *at the leaf nodes as a function of the parameter ^*k*^*t *∈ TK for each topology label *k *∈ K. Such a model prescribes $P_{a_{i},a_{j}}(t)$, the probability of mutation from a character *a*_*i *_∈ U to another character *a*_*j *_∈ U in time *t*. Using such a transition probability we may compute ℓ_*q*_(^*k*^*t*), the log-likelihood of the data *d *at site *q *∈ {1,..., *v*} or the *q*-th column of *d*, via the post-order traversal over the labeled tree with branch lengths ^*k*^*t *:= (^*k*^*t*_1_, ^*k*^*t*_2_,...,tkbk). This amounts to the sum-product Algorithm 2 [16] that associates with each node *h *∈ {1,..., *s*_*k*_} of ^*k*^*t *subtending ℏ many descendants, a partial likelihood vector, $l_{h}:=(l_{h}^{\left(a_{1}\right)},l_{h}^{\left(a_{2}\right)},...,l_{h}^{\left(a_{\left|U\right|}\right)})\in {\mathbb{R}}^{\left|U\right|}$, and specifies the length of the branch leading to its ancestor as ^*k*^*t*_*h*_.

**input **: (i) a labeled tree with branch lengths ^*k*^*t *:= (^*k*^*t*_1_, ^*k*^*t*_2_,...,tkbk), (ii) transition probability $P_{a_{i},a_{j}}(t)$ for any *a*_*i*_, *a*_*j *_∈ U, (iii) stationary distribution *π*(*a*_*i*_) over each character *a*_*i *_∈ U, (iv) site pattern or data *d*_•, *q *_at site *q*

**output **: $l_{d_{\cdot ,q}}$(^*k*^*t*), the likelihood at site *q *with pattern *d*_•, *q*_

**initialize**: For a leaf node *h *with observed character *a*_*i *_= *d*_*h*, *q *_at site *q*, set $l_{h}^{\left(a_{i}\right)}$ = 1 and $l_{h}^{\left(a_{j}\right)}$ = 0 for all *j *≠ *i*. For any internal node *h*, set *l*_*h *_:= (1, 1,...,1).

**recurse **: compute *l*_*h *_for each sub-terminal node *h*, then those of their ancestors recursively to finally compute *l*_*r *_for the root node *r *to obtain the likelihood for site *q*,

ld·,q(tk)=lr=∑ai∈U(π(ai)⋅lr(ai)).

For an internal node *h *with descendants *s*_1_, *s*_2_,..., *s*_ℏ_,

lh(ai)=∑j1,...,jℏ∈U{ls1(j1)⋅Pai,j1( kts1)⋅ls2(j2)⋅Pai,j2( kts2)…lsℏ(jℏ)⋅Pai,jℏ( ktsℏ)}.

**Algorithm 2**: Likelihood by post-order traversal

Assuming independence across all *v *sites we obtain the likelihood function for the given data *d*, by multiplying the site-specific likelihoods

(2) ld(tk)=∏q=1vld·,q(tk).

The maximum likelihood estimate is a point estimate (single best guess) of the unknown phylogenetic tree on the basis of the observed data *d *and it is

arg⁡max⁡tk∈TKld(tk).

The simplest probability models for character mutation are continuous time Markov chains with finite state space U. We introduce three such models employed in this study next. We only derive the likelihood functions for the simplest model with just two characters as it is thought to well-represent the core problems in phylogenetic estimation (see for e.g. [17]).

#### Posterior density of a tree

The posterior density *f*^• ^(^*k*^*t*) conditional on data *d *at tree ^*k*^*t *is the normalized product of the likelihood *l*_*d*_(^*k*^*t*) and the prior density *p*(^*k*^*t*) over a given tree space TK:

(3) f·(tk)=ld(tk)p(tk)∫KTld(tk)p(tk)∂(tk).

We assume a uniform prior density over a large box or a union of large boxes in a given tree space TK. Typically, the sides of the box giving the range of branch lengths, are extremely long, say, [0, 10] or [10^-10^, 10]. The branch lengths are measured in units of expected number of DNA substitutions per site and therefore the support of our uniform prior density over TK contains the biologically relevant branch lengths. If TK is a union of distinct topologies then we let our prior be an equally weighted finite mixture of uniform densities over large boxes in each topology. Naturally, other prior densities are possible especially in the presence of additional information. We choose at priors for the convenient interpretation of the target posterior shape f(tk)=f·(tk)∫TKld(tk)p(tk)∂(tk) to be the likelihood function in the absence of prior information beyond a compact support specification.

#### Likelihood of a triplet under Cavender-Farris-Neyman (CFN) model

We now describe the simplest model for the evolution of binary sequences under a symmetric transition matrix over all branches of a tree. This model has been used by authors in various fields including molecular biology, information theory, operations research and statistical physics; for references see [7,18]. This model is referred to as the Cavender-Farris-Neyman (CFN) model in molecular biology, although in other fields it has been referred to as 'the on-off machine', 'symmetric binary channel' and the 'symmetric two-state Poisson model'. Although the relatively tractable CFN model itself is not popular in applied molecular evolution, the lessons learned under the CFN model often extend to more realistic models of DNA mutation (e.g. [17]). Thus, our first stop is the CFN model.

**Model 1 (Cavender-Farris-Neyman (CFN) model) ***Under the CFN mutation model, only pyrimidines and purines, denoted respectively by *Y:= {C, T} *and *R:= {A, G}, *are distinguished as evolutionary states among the four nucleotides *{A, G, C, T}, *i.e*. U = {Y, R}. *Time t is measured by the expected number of substitutions in this homogeneous continuous time Markov chain with rate matrix:*

$$Q=\left(\begin{array}{cc} -1 & 1\\ 1 & -1 \end{array}\right),$$

*and transition probability matrix P*(*t*) = *e*^*Qt *^:

$$P(t)=\left(\begin{array}{cc} 1-(1-e^{-2t})/2 & (1-e^{-2t})/2\\ (1-e^{-2t})/2 & 1-(1-e^{-2t})/2 \end{array}\right).$$

*Thus, the probability that *Y *mutates to *R, *or vice versa, in time t is a*(*t*): (1*e*^-2*t*^)/2. *The stationary distribution is uniform on *U, *i.e*. *π*(R) = *π*(Y) = 1/2.

When there are only three taxa, there are five tree topologies of interest as depicted in Figure 1. There are 2^3 ^= 8 possible site patterns, i.e. for each site *q *∈ {1, 2,..., *v*}, the *q*-th column of the data *d*, denoted by *d*_•, *q*_, is one of eight possibilities, numbered 0, 1,...,7 for convenience:

(4) $$d_{\cdot ,q}\in \left\{\begin{array}{ccccccccccccccc} 0 & , & 1 & , & 2 & , & 3 & , & 4 & , & 5 & , & 6 & , & 7\\ \text{R} & & \text{Y} & & \text{R} & & \text{Y} & & \text{R} & & \text{Y} & & \text{R} & & \text{Y}\\ \text{R} & , & \text{Y} & , & \text{R} & , & \text{Y} & , & \text{Y} & , & \text{R} & , & \text{Y} & , & \text{R}\\ \text{R} & & \text{Y} & & \text{Y} & & \text{R} & & \text{Y} & & \text{R} & & \text{R} & & \text{Y} \end{array}\right\}.$$

Given a multiple sequence alignment data *d *from 3 taxa at *v *homologous sites, i.e. *d *∈ {Y, R}^3 × *v*^, the likelihood function over the tree space Tk is simplified from (2) as follows:

(5) ld(tk)=∏q=1vld·,q(tk)=∏i=07(li(tk))ci,

where *l*_*i*_(^*k*^*t*) is the likelihood of the the *i*-th site pattern as in (4) and *c*_*i *_is the count of sites with pattern *i*. In fact, *l*_*i*_(^*k*^*t*) = *P*(*i*|^*k*^*t*) is the probability of observing site pattern *i *given topology label *k *and branch lengths *t *and similarly *l*_*d*_(^*k*^*t*) = *P*(*d*|^*k*^*t*).

Consider the unrooted tree-space with a single topology labeled 4 and three non-negative terminal branch lengths ^4^*t *= (^4^*t*_1_, ^4^*t*_2_, ^4^*t*_3_) ∈ ${\mathbb{R}}_{+}^{3}$ as shown in Figure 1(v). An application of Algorithm 2 to compute the likelihoods *l*_0_(^4^*t*), *l*_1_(^4^*t*),..., *l*_7_(^4^*t*), as derived in (19)-(25), reveals symmetry. There are in fact four minimally sufficient site pattern classes, namely, xxx, xxy, yxx and xyx, where x and y simply denote distinct characters in the alphabet set U = {R, Y}. The corresponding likelihoods are:

(6) lxxx(t4):=l0(t4)=l1(t4)=18(1+e−2( 4t1+ 4t2)+e−2( 4t2+ 4t3)+e−2( 4t1+ 4t3))lxxy(t4):=l2(t4)=l3(t4)=18(1+e−2( 4t1+ 4t2)−e−2( 4t2+ 4t3)−e−2( 4t1+ 4t3))lyxx(t4):=l4(t4)=l5(t4)=18(1−e−2( 4t1+ 4t2)+e−2( 4t2+ 4t3)−e−2( 4t1+ 4t3))lxyx(t4):=l6(t4)=l7(t4)=18(1−e−2( 4t1+ 4t2)−e−2( 4t2+ 4t3)+e−2( 4t1+ 4t3)).

Therefore, the multiple sequence alignment data *d *from three taxa evolving under Model 1 can be summarized by the minimal sufficient site pattern counts

(*c*_xxx_, *c*_xxy_, *c*_yxx_, *c*_xyx_):= (*c*_0 _+ *c*_1_, *c*_2 _+ *c*_3_, *c*_4 _+ *c*_5_, *c*_6 _+ *c*_7_),

which simplifies (5) to:

(7) ld(tk)=∏q=1vld·,q(tk)=∏i=07(li(tk))ci=∏s=xxx,xxy,yxx,xyx(ls(tk))cs.

Note that the probability of our sample space with eight patterns given in (4) is ∑i=07li(t4)=1. Our likelihoods are half of those in [17] that are prescribed over a sample space of only four classes of patterns: {0, 1}, {2, 3}, {4, 5} and {6, 7}. This is because we distinguish between the sample space of data from that of the minimal sufficient statistics. We compute the rooted topology-specific likelihood functions, i.e. *l*(^*k*^*t*) for *k *∈ {0, 1, 2, 3} (Figure 1) by substituting the appropriate constraints on branch lengths in T4=ℝ+3, the space of unrooted triplets.

#### Likelihood of a triplet under Jukes-Cantor (JC) model

The *r*-state symmetric model introduced in [19] is specified by the *r *× *r *rate matrix with equal off-diagonal entries over an alphabet set U of size *r*. The stationary distribution under this model is the uniform distribution on U. Thus, CFN model is the 2-state symmetric model over U = {Y, R}. The Jukes-Cantor (JC) model [20] is the 4-state symmetric model over U = {A, C, G, T}. This is perhaps the simplest model on four characters.

**Model 2 (Jukes-Cantor (JC) model) ***All four nucleotides form the state space for this mutation model, i.e*. U = {A, C, G, T}. *Once again, evolutionary time t is measured by the expected number of substitutions in the homogeneous continuous time Markov chain with rate matrix:*

$$Q=\left(\begin{array}{cccc} -1 & 1/3 & 1/3 & 1/3\\ 1/3 & -1 & 1/3 & 1/3\\ 1/3 & 1/3 & -1 & 1/3\\ 1/3 & 1/3 & 1/3 & -1 \end{array}\right).$$

*The transition probability matrix P*(*t*) = *e*^*Qt *^*is also symmetric. The probability that any given nucleotide mutates to any other nucleotide in time t is P*_x, y_(*t*) *and that it is found in the same state is P*_x, x_(*t*). *These transition probabilities are:*

$$\begin{array}{cc} a(t):=P_{\text{x},\text{y}}(t)=\frac{1}{4}-\frac{1}{4}\exp\left(-\frac{4}{3}t\right), & b(t):=P_{\text{x},\text{x}}(t)=\frac{1}{4}+\frac{3}{4}\exp\left(-\frac{4}{3}t\right). \end{array}$$

*The stationary distribution is uniform, i.e*. *π*(A) = *π*(C) = *π*(G) = *π*(T) = 1/4.

Consider the three non-negative terminal branch lengths ^4^*t *= (^4^*t*_1_, ^4^*t*_2_, ^4^*t*_3_) ∈ ${\mathbb{R}}_{+}^{3}$ of an unrooted tree ^4^*t *of Figure 1(v). An application of Algorithm 2 to compute the likelihoods of the 64 possible site patterns (see for e.g. [21-24]), reveals five minimally sufficient site pattern classes. Let x, y and z simply denote distinct characters from the alphabet set U = {A, C, G, T} at taxon 1, 2 and 3, respectively. The minimally sufficient site pattern classes xxx, xyz, xxy, yxx and xyx encode 4, 24, 12, 12 and 12 nucleotide site patterns, respectively. By a computation similar to that in (19)-(25), the likelihoods are:

lxxx(t4)=14(∏i=13b( 4ti)+3∏i=13a( 4ti))lxyz(t4)=14(b( 4t1)a( 4t2)a( 4t3)+a( 4t1)(b( 4t2)a( 4t3)+a( 4t2)(b( 4t3)+a( 4t3))))lxxy(t4)=14(b( 4t1)b( 4t2)a( 4t3)+a( 4t1)a( 4t2)(b( 4t3)+2a( 4t3)))lxyx(t4)=14(b( 4t1)a( 4t2)b( 4t3)+a( 4t1)a( 4t3)(b( 4t2)+2a( 4t2)))lyxx(t4)=14(a( 4t1)b( 4t2)b( 4t3)+a( 4t2)a( 4t3)(b( 4t1)+2a( 4t1))).

Notice that the probability of observing one of the 64 possible site patterns is 1 for any ^4^*t *∈ (0, ∞)^3 ^:

4*l*_xxx_(^4^*t*) + 24*l*_xyz_(^4^*t*) + 12*l*_xxy_(^4^*t*) + 12*l*_yxx_(^4^*t*) + 12*l*_yxx_(^4^*t*) = 1.

Let *c*_ijk _denote the number of sites with the site pattern ijk ∈ {xxx, xyz, xxy, yxx, xyx}. Then, under the assumption of independence across sites, we obtain the likelihood of a given data *d *by multiplying the site-specific likelihoods:

ld(t4)=(lxyz(t4))cxyz(lxxy(t4))cxxy(lxyx(t4))cxyx(lyxx(t4))cyxx(lxxx(t4))cxxx.

Once again, the likelihood of a rooted tree or the star tree can be obtained from that of the unrooted tree by substituting the appropriate constraints on branch lengths in the above equations or by directly applying Algorithm 2 with the appropriate input tree with its topology and branch lengths.

**Model 3 (Hasegawa-Kishino-Yano (HKY) model) ***The Hasegawa-Kishino-Yano or HKY model *[25]*has all four nucleotides in the state space, i.e*. U = {A, C, G, T}. *There are five parameters in this more flexible model. Transitions are changes within the purine *{A, G} *or pyrimidine *{C, T} *state subsets, while transversions are changes from purine to pyrimidine or from pyrimidine to purine. In this model, we have a mutational parameter κ that allows for transition:transversion bias and four additional parameters π*_A_, *π*_C_, *π*_G _*and π*_T _*that explicitly control the stationary distribution. The entries of the rate matrix are:*

$$q_{x,y}=\{\begin{array}{ll} \kappa \pi_{y} & for\;transitions\\ \pi_{y} & for\;transversions\\ -\sum_{z\in U,z\neq x}q_{x,z} & if\;x=y. \end{array}$$

*The transition probabilities are known analytically for this model (see for e.g*. [[8], *p. 203]). We can use these expressions when evaluating the likelihood of a rooted or unrooted tree along with the five mutational parameters via Algorithm 2. For simplicity we set the stationary distribution parameters to the empirical nucleotide frequencies and κ to be *2.0 *in this study*.

### Interval analysis

Let Iℝ denote the set of closed and bounded real intervals. Let any element of Iℝ be denoted by ***x***: [$\underline{x}$, $\bar{x}$], where, $\underline{x}$ ≤ $\bar{x}$ and $\underline{x}$, $\bar{x}$ ∈ ℝ. Next we define arithmetic over Iℝ.

**Definition 1 (Interval Operation) ***If the binary operator ⋆ is one of *+, -, ×,/, *then we define an arithmetic on operands in *Iℝ* by*

***x ***⋆ ***y***:= {*x *⋆ *y *: *x *∈ ***x***, *y *∈ ***y***},

*with the exception that ****x***/***y ****is undefined if *0 ∈ ***y***.

**Theorem 1 (Interval arithmetic) ***Arithmetic on the pair ****x***, ***y ***∈ Iℝ*is given by:*

$$\begin{array}{lll} x+y & = & \left[\underline{x}+\underline{y},\bar{x}+\bar{y}\right]\\ x-y & = & \left[\underline{x}-\bar{y},\bar{x}-\underline{y}\right]\\ x\times y & = & \left[\min\{\underline{x}\underline{y},\underline{x}\bar{y},\bar{x}\underline{y},\bar{x}\bar{y}\},\max\{\underline{x}\underline{y},\underline{x}\bar{y},\bar{x}\underline{y},\bar{x}\bar{y}\}\right]\\ x/y & = & x\times [1/\bar{y},1/\underline{y}],\;provided,\;0\notin y. \end{array}$$

When computing with finite precision, say in floating-point arithmetic, directed rounding must be taken into account (see e.g., [6,10]) to contain the solution. Interval multiplication is branched into nine cases, on the basis of the signs of the boundaries of the operands, such that only one case entails more than two real multiplications. Therefore, a rigorous computer implementation of an interval operation mostly requires two directed rounding floating-point operations. Interval addition and multiplication are both commutative and associate but not distributive. For example,

$$\begin{array}{c} {}[-1,2]\times ([1,2]+[-2,1])=[-1,2]\times [-1,3]=[-3,6],\\ \begin{array}{cc} \text{but}, & [-1,2]\times [1,2]|[-1,2]\times [-2,1]=[-2,4]+[-4,2]=[-6,6]. \end{array} \end{array}$$

Interval arithmetic satisfies a weaker rule than distributivity called sub-distributivity:

***x***(***y ***+ ***z***) ⊆ ***xy ***+ ***xz***.

An extremely useful property of interval arithmetic that is a direct consequence of Definition 1 is summarized by the following theorem.

**Theorem 2 (Fundamental property of interval arithmetic) ***If ****x ***⊆ ***x***' *and ****y ***⊆ ***y***' *and *⋆ ∈ {+, -, ×,/}, *then*

***x ***⋆ ***y ***⊆ ***x***' ⋆ ***y***',

*where we require that *0 ∉ ***y***' *when *⋆ = /.

Note that an immediate implication of Theorem 2 is that when ***x ***= [*x*, *x*] and ***y ***= [*y*, *y*] are thin intervals, i.e. $\underline{x}$ = $\bar{x}$ = *x *and $\underline{y}$ = $\bar{y}$ = *y *are real numbers, then ***x***' ⋆ ***y***' will contain the result of the real arithmetic operation *x *⋆ *y*.

Let $\underline{x}$ , $\bar{x}$ ∈ ℝ^*n *^be real vectors such that $\underline{x}$_*i *_≤ ${\bar{x}}_{i}$, for all *i *= 1, 2,..., *n*, then ***x ***: [$\underline{x}$ , $\bar{x}$] is an *interval vector *or a *box*. The set of all such boxes is $I{\mathbb{R}}^{n}$. The *i*-th component of the box ***x ***= (***x***_1_,..., ***x***_*n*_) is the interval ***x***_*i *_= [$\underline{x}$_*i*_, ${\bar{x}}_{i}$] and the interval extension of a set $D\subseteq {\mathbb{R}}^{n}$ is $ID:=\{x\in I{\mathbb{R}}^{n}:\underline{x},\bar{x}\in D\}$. We write inf ***x ***:= $\underline{x}$ for the *lower bound*, sup ***x ***:= $\bar{x}$ for the *upper bound*. Let the maximum norm of a vector *x *∈ ℝ^*n *^be ∥*x*∥_∞ _:= max_*k *_|*x*_*k*_|. Let the vector valued hyper-metric between boxes ***x ***and ***y ***be

$$\text{dist}(x,y)=\sup\{|\underline{x}-\underline{y}|,|\bar{x}-\bar{y}|\},$$

and the Hausdorff distance between the boxes ***x ***and ***y ***in the metric given by the maximum norm is then

dist_∞ _(***x***, ***y***) = ∥dist(***x***, ***y***)∥_∞_.

We can make $I{\mathbb{R}}^{n}$ a metric space by equipping it with the Hausdorff distance.

Our main motivation for the extension to intervals is to enclose the *range*:

range(*f*; *S*):= {*f*(*x*): *x *∈ *S*},

of a real-valued function *f *: ℝ^*n *^↦ ℝ over a set *S *⊆ ℝ^*n*^. Except for trivial cases, few tools are available to obtain the range.

**Definition 2 (Directed acyclic graph (DAG) expression of a function) ***One can think of the process by which a function f *: ℝ^*m *^↦ ℝ *is computed as the result of a sequence of recursive operations with the sub-expressions *f_*i *_*of its expression *f *where, i *= 1,..., *n *< ∞. *This involves the evaluation of the sub-expression *f_*i*_* at node i with operands *$s_{i_{1}}$, $s_{i_{2}}$*from the sub-terminal nodes of i given by the directed acyclic graph (DAG) for *f

(8) $$s_{i}=⊙{\text{f}}_{i}:=\{\begin{array}{ll} {\text{f}}_{i}(s_{i_{1}},s_{i_{2}}) & :if\;node\;i\;has\;2\;sub\text{-}terminal\;nodes\;s_{i_{1}},s_{i_{2}}\\ {\text{f}}_{i}(s_{i_{1}}) & :if\;node\;i\;has\;1\;sub\text{-}terminal\;node\;s_{i_{1}}\\ I(s_{i}) & :if\;node\;i\;is\;a\;leaf\;or\;terminal\;node,I(x)=x. \end{array}$$

*The leaf or terminal node of the DAG is a constant or a variable and thus the *f_*i *_*for a leaf i is set equal to the respective constant or variable. The recursion starts at the leaves and terminates at the root of the DAG. The DAG for an elementary f is simply its expression *f *with n sub-expressions *f_1_, f_2_,...,f_*n*_:

(9) $$\begin{array}{ccc} {\left\{⊙{\text{f}}_{i}\right\}}_{i=1}^{n} & ↣ & ⊙{\text{f}}_{n}=\text{f}(x), \end{array}$$

*where each *⊙f_*i *_*is computed according to *(8).

We look at some DAGs for 0 functions to concretely illustrate these ideas.

**Figure 2:**
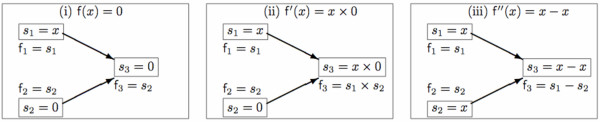
**DAG expression for zero functions**. The directed acyclic graph (DAG) expression for the three zero functions: (i) f(*x*) = 0, (ii) f'(*x*) = *x *× 0 and (iii) f"(*x*) = *x *- *x*.

**Example 1 ***Consider the constant zero function f*(*x*) = 0 *expressed as (i) *f(*x*) = 0, *(ii) *f'(*x*) = *x *× 0 *and (iii) *f"(*x*) = *x *- *x. The corresponding DAG expressions are shown in Figure *2.

**Definition 3 (The natural interval extension) ***Consider a real-valued function f*(*x*): ℝ^*n *^↦ ℝ^*m*^* given by a formula or a DAG expression *f(*x*). *If real constants, variables, and operations in *f(*x*) *are replaced by their interval counterparts, then one obtains*

$$f(x):I{\mathbb{R}}^{n}\mapsto I{\mathbb{R}}^{m}.$$

**f**(*x*) *is known as the natural interval extension of the expression *f(*x*) *for f*(*x*). *This extension is well-defined if we do not run into division by zero*.

Although the three distinct expressions f(*x*), f'(*x*) and f"(*x*) of the real function *f*: ℝ ↦ ℝ of Example 1 are equivalent upon evaluation in the reals, their respective interval extensions **f**(***x***) = [0, 0], **f'**(***x***) = ***x ***× [0, 0], and **f"**(***x***) = ***x ***- ***x ***are not. For instance, if ***x ***= [1, 2],

$$\begin{array}{lll} f([1,2]) & = & [0,0],\\ f^{\prime }([1,2]) & = & \left[1,2]\times [0,0]=[\min\{1\times 0,1\times 0,2\times 0,2\times 0\},\max\{1\times 0,1\times 0,2\times 0,2\times 0\}]=[0,0\right]\\ f^{\prime \prime }([1,2]) & = & [1,2]-[1,2]=[1-2,2-1]=[-1,1], \end{array}$$

and in general for any ***x ***: [$\underline{x}$ , $\bar{x}$] ∈ Iℝ,

$$\begin{array}{lll} f([\underline{x},\bar{x}]) & = & [0,0],\\ f^{\prime }([\underline{x},\bar{x}]) & = & \left[\underline{x},\bar{x}]\times [0,0]=[\min\{\underline{x}\times 0,\underline{x}\times 0,\bar{x}\times 0,\bar{x}\times 0\},\max\{\underline{x}\times 0,\underline{x}\times 0,\bar{x}\times 0,\bar{x}\times 0\}]=[0,0\right]\\ f^{\prime \prime }([\underline{x},\bar{x}]) & = & \begin{array}{cc} {}[\underline{x},\bar{x}]-[\underline{x},\bar{x}]=[\underline{x}-\bar{x},\bar{x}-\underline{x}]\neq [0,0], & \text{unless }\underline{x}=\bar{x}. \end{array} \end{array}$$

Thus, **f**(***x***) = **f'**(***x***) ≠ **f"**(***x***) for any ***x ***∈ Iℝ, albeit f(*x*) = f'(*x*) = f"(*x*) for any *x *∈ ℝ.

**Theorem 3 (Interval rational functions) ***Consider the rational function f*(*x*) = *p*(*x*)/*q*(*x*), *where p and q are polynomials. Let ***f ***be the natural interval extension of its DAG expression *f *such that ***f**(***y***) *is well-defined for some ****y *∈ **Iℝ* and let ****x***, ***x' ***∈ Iℝ. *Then we have*

$$\begin{array}{lll} \left(i\right) & Inclusion\;isotony: & \begin{array}{cc} \forall x\subseteq x^{\prime }\subseteq y\Rightarrow f(x)\subseteq f(x^{\prime }), & and \end{array}\\ \left(ii\right) & Range\;enclosure: & \forall x\subseteq y\Rightarrow \text{range}(f;x)\subseteq f(x). \end{array}$$

**Definition 4 (Standard functions) ***Piece-wise monotone functions, including exponential, logarithm, rational power, absolute value, and trigonometric functions, constitute the set of standard functions*

S = {*a*^*x*^, *log*_*b*_(*x*), *x*^*p*/*q*^, |*x*|, sin(*x*), cos(*x*), tan(*x*), sinh(*x*),...arcsin(*x*),...}.

Such functions have well-defined interval extensions that satisfy inclusion isotony and *exact range enclosure*, i.e. range(*f*; ***x***) = **f**(***x***). Consider the following definitions for the interval extensions for some monotone functions in S with ***x ***∈ Iℝ,

$$\begin{array}{llll} \exp(x) & = & \left[\exp(\underline{x}),\exp(\bar{x})\right] & \\ \arctan(x) & = & \left[\arctan(\underline{x}),\arctan(\bar{x})\right] & \\ \sqrt{\left(x\right)} & = & \left[\sqrt{\left(\underline{x}\right)},\sqrt{\left(\bar{x}\right)}\right] & \text{if }0\leq \underline{x}\\ \log(x) & = & \left[\log(\underline{x}),\log(\bar{x})\right] & \text{if }0<\underline{x}, \end{array}$$

and a piece-wise monotone function in S; with ℤ_+ _and ℤ_- _representing the set of positive and negative integers, respectively. Let the *mignitude *of an interval ***x ***be the number 〈x〉 = min{|*x*|:*x *∈ ***x***} and the *absolute value *of ***x ***be the number |***x***| = max{|*x*|:*x *∈ ***x***} = sup{-$\underline{x}$ , $\bar{x}$}. Then, the interval-extended power function that plays a basic role in product likelihood functions is:

$$x^{n}=\{\begin{array}{ll} \left[{\underline{x}}^{n},{\bar{x}}^{n}\right] & :\text{if }n\in {\mathbb{Z}}_{+}\text{ is odd},\\ \left[{\langle x\rangle }^{n},|x{|}^{n}\right] & :\text{if }n\in {\mathbb{Z}}_{+}\text{ is even},\\ \left[1,1\right] & :\text{if }n=0,\\ {\left[1/\bar{x},1/\underline{x}\right]}^{-n} & :\text{if }n\in {\mathbb{Z}}_{-}\text{; 0}\notin x. \end{array}$$

**Definition 5 (Elementary functions) ***A real-valued function that can be expressed as a finite combination of constants, variables, arithmetic operations, standard functions and compositions is called an elementary function. The set of all such elementary functions is referred to as *E.

**Example 2 (Probability of the pattern **xxx **under CFN star tree **^0^*t***) ***The trifurcating star-tree *^0^*t *:= (^0^*t*_1_) *has topology label *0 *and common branch length parameter *^0^*t*_1 _*as shown in *Figure 1(i). *Either a direct application of Algorithm 2 with input as *^0^*t *:= (^0^*t*_1_) *or a substitution of *^0^*t*_1 _*for *^4^*t*_1_, ^4^*t*_2 _*and *^4^*t*_3 _*in *(6), *yields the likelihood for pattern *xxx *as:*

lxxx(t0)=(1+3e−4( 0t1))/8.

**Figure 3:**
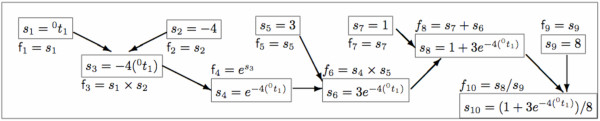
**DAG expression for probability of the pattern xxx under a CFN star tree**. The elementary function l0(t0)=(1+3e−4( 0t1))/8. can be obtained from the terminus ⊙f_10 _of the recursion ${\left\{⊙f_{i}\right\}}_{i=1}^{10}$ over the sub-expressions f_1_,...,f_10 _in the above directed acyclic graph (DAG) expression of *l*_0_(^0^*t*). Note that the leaf nodes are constants (*s*_2_, *s*_5_, *s*_7 _and *s*_9_) or variables (*s*_1_).

*The probability of the pattern *xxx *under CFN star tree *^0^*t given by l*_xxx_(^0^*t*) *with the corresponding DAG expression shown in Figure *3* is an elementary function*.

It would be convenient if guaranteed enclosures of the range of an elementary *f *can be obtained by the natural interval extension **f **of one of its expressions f. The following Theorem 4 is the work-horse of interval Monte Carlo algorithms.

**Theorem 4 (The fundamental theorem of interval analysis) ***Consider any elementary function f *∈ E* with expression *f. *Let ***f **: ***y ***↦ Iℝ* be its natural interval extension such that ***f**(***y***) *is well-defined for some ****y *∈ **Iℝ* and let ****x***, ***x' ***∈ Iℝ. *Then we have*

$$\begin{array}{llll} \left(i\right) & Inclusion\;isotony: & \forall x\subseteq x^{\prime }\subseteq y\Rightarrow f(x)\subseteq f(x^{\prime }), & and\\ \left(ii\right) & Range\;enclosure: & \forall x\subseteq y\Rightarrow \text{range}(f;x)\subseteq f(x). & \end{array}$$

The fundamental implication of the above theorem is that it allows us to enclose the range of any elementary function and thereby produces an upper bound for the global maximum and a lower bound for the global minimum over any compact subset of the domain upon which the function is well-defined. This is the work-horse for rigorously constructing an envelope for rejection sampling.

**Figure 4:**
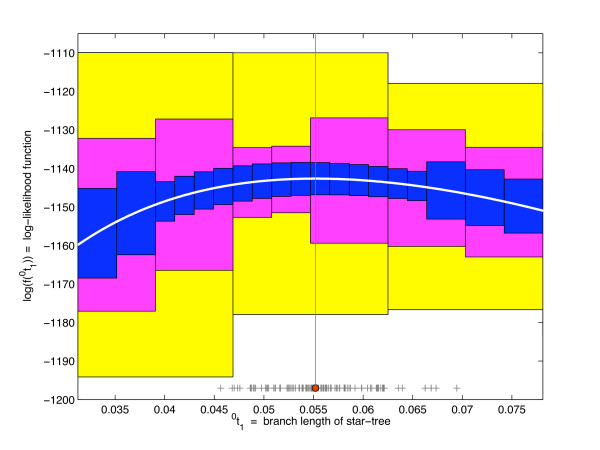
**Adaptive range enclosure of the posterior density over the star-tree space**. Range enclosure of the log-likelihood (white line) for the human, chimpanzee and gorilla mitochondrial sequence data [27] analyzed in [17], under the CFN model with *c*_xxx _= 762 and *v *= 895 over star-trees, via its interval extension linearly tightens with the mesh. One hundred samples (+) from the MRS and the maximum likelihood estimate (red dot) are shown.

Unlike the natural interval extension of an *f *∈ S that produces exact range enclosures, the natural interval extension **f**(***x***) of an *f *∈ E often overestimates range(*f*; ***x***), but can be shown under mild conditions to linearly approach the range as the maximal width of the box ***x ***goes to zero. This implies that a partition of ***x ***into smaller boxes {***x***^(1)^,⋯, ***x***^(*m*)^} gives better enclosures of range(*f*; ***x***) through the union $\cup_{i=1}^{m}f(x^{\left(i\right)})$ as illustrated in Figure 4. Next we make the above statements precise in terms of the *width *and *radius *of a box ***x ***defined by wid ***x ***:= $\bar{x}$ - $\underline{x}$ and rad ***x ***:= ($\bar{x}$ - $\underline{x}$ )/2, respectively.

**Definition 6 ***A function f*: D ↦ ℝ *is Lipschitz if there exists a Lipschitz constant K such that, for all x, y *∈ D, *we have *|*f*(*x*) - *f*(*y*)| ≤ *K*|*x *- *y*|. *We define *$E_{L}$* to be the set of elementary functions whose sub-expressions *f_*i*_, *i *= 1,..., *n at the nodes of its corresponding DAG *f *are all Lipschitz:*

$$E_{L}:=\{f\in E:each\;sub\text{-}expression{\text{ f}}_{i}\;in\;the\;DAG\;expression\text{ f }for\;f\;is\;Lispschitz\}.$$

**Theorem 5 (Range enclosure tightens linearly with mesh) ***Consider a function f *: D ↦ ℝ*with f *∈ $E_{L}$. *Let ***f ***be an inclusion isotonic interval extension of the DAG expression *f *of f such that ***f **(***x***) *is well-defined for some ****x ***∈ Iℝ. *Then there exists a positive real number K, depending on ***f ***and ****x***, *such that if *$x=\cup_{i=1}^{k}x^{\left(i\right)}$, *then*

$$\text{range}(f;x)\subseteq \overset{k}{\underset{i=1}{\cup }}f(x^{\left(i\right)})\subseteq f(x),$$

and

$$\text{rad}\left(\overset{k}{\underset{i=1}{\cup }}f(x^{\left(i\right)})\right)\leq \text{rad }(\text{range}(f;x))+K\underset{i=1,...,k}{\max}\text{rad}(x^{\left(i\right)}).$$

#### Likelihood of a box of trees

The likelihood function (2) over trees with a DAG expression that is directly or indirectly obtained via Algorithm 2 has a natural interval extension over boxes of trees [5,26]. This interval extension of the likelihood function allows us to produce rigorous enclosures of the likelihood over a box in the tree space. Next we give a concrete example of the natural interval extension of the likelihood function over an interval of trees ^0^***t ***in the star-tree space  0T. The same ideas extend to any labeled box of trees ^*k*^***t ***when the number of branch lengths is greater than one and more generally to a finite union of labeled boxes with possibly distinct labels.

**Example 3 (Posterior density over the CFN star-tree space ** 0T) *The trifurcating star-tree *^0^*t *:= (^0^*t*_1_) *has topology label *0 *and common branch length *^0^*t*_1 _> 0. *Either a direct application of Algorithm 2 with input triplet *^0^*t or a substitution of *^4^*t*_1_, ^4^*t*_2 _*and *^4^*t*_3 _*in *(6) *by *^0^*t*_1 _*yields the following * 0T-*specific likelihoods:*

(10) l0(t0)=l1(t0)=(1+3e−4( 0t1))/8,l2(t0)=l3(t0)=l4(t0)=l5(t0)=l6(t0)=l7(t0)=(1−e−4( 0t1))/8.

*Therefore, on the basis of *(4), (5), (6) *and *(7), *the likelihood of the data at the star-tree *^0^*t *∈  0T*is*

(11) ld(t0)=∏i=07(li(t0))ci=((1+3e−4( 0t1))/8)c0+c1((1−e−4( 0t1))/8)∑i=27ci=((1+3e−4( 0t1))/8)c0+c1((1−e−4( 0t1))/8)v−(c0+c1),

*the posterior density *(3) *based on a uniform prior p*(^0^*t*_1_) = 1/10 *over * 0T = (0, 10] *is*

(12) f·(t0)=ld(t0)∫010ld(t0)∂(t0).

*Thus, under our conveniently chosen uniform prior, the target posterior shape (without the normalizing constant) is simply the likelihood function, i.e*.

f(t0)=f·(t0)∫010ld(t0)∂(t0)=ld(t0).

*Observe that the minimal sufficient statistics over * 0T*are the number of sites with the same character c*_xxx _:= *c*_0 _+ *c*_1 _*and the total number of sites v. Let the natural interval extension of the DAG expression for the posterior shape f*(^0^*t*):  0T ↦ ℝ *be:*

f(t0):I0T↦Iℝ.

*Thus*, **f ***maps an interval *^0^***t ****in the tree space * 0T*to an interval in *Iℝ*that encloses the target shape or likelihood of *^0^***t***.

*For the human, chimpanzee and gorilla mitochondrial sequence data *[27]*analyzed in *[17], *c*_xxx _= 762 *and v *= 895. Figure 4* shows *log(*f*(^0^*t*)) *or the log-likelihood function for this data set as the white line. Evaluations of its interval extension over partitions by 3, 7 and 19 intervals are depicted by colored rectangles in *Figure 4. *Notice how the range enclosure by the interval extension of the log-likelihood function, our target shape, tightens with domain refinement as per Theorem 5. The maximum likelihood estimate derived in *[17]*(the red dot in *Figure 4*) is*

t0^=arg⁡max⁡t0∈T0f(t0)=(0.055205).

### Moore rejection sampler (MRS)

Moore rejection sampler (MRS) is an auto-validating rejection sampler (RS). MRS is said to be auto-validating because it automatically obtains a proposal *g *that is easy to simulate from, and an envelope $\hat{g}$ that is guaranteed to satisfy the envelope condition (1). MRS can produce independent samples from any target shape *f *whose DAG expression f has a well-defined natural interval extension **f **over a compact domain T. In summary, the defining characteristics and notations of MRS are:

$$\begin{array}{ll} \text{Compact domain} & T=[\underline{t},\bar{t}]\\ \text{Target shape} & f(t):T\mapsto \mathbb{R}\\ \text{Target integral} & N_{f}:=\int_{T}f(t)dt\\ \text{Target density} & f^{\cdot }(t):={\left(N_{f}\right)}^{-1}f(t):T\mapsto \mathbb{R}\\ \text{DAG expression of }f & \text{f}(t):T\mapsto \mathbb{R}\\ \text{Interval extension of f} & f(t):IT\mapsto I\mathbb{R}\\ \text{Envelope function} & \hat{g}(t):T\mapsto \mathbb{R}\\ \text{Envelope integral} & N_{\hat{g}}:=\int_{T}\hat{g}(t)dt\\ \text{Proposal density} & g(t):={\left(N_{\hat{g}}\right)}^{-1}\hat{g}(t):T\mapsto \mathbb{R}\\ \text{Acceptance probability} & A(\hat{g})=N_{f}/N_{\hat{g}}\\ \text{Partitionof }T & T:=\{t^{\left(1\right)},t^{\left(2\right)},...,t^{\left(|T|\right)}\}. \end{array}$$

Suppose *f *is an elementary function and its DAG expression f has a well-defined interval extension **f **on T. If $T:=\{t^{\left(1\right)},t^{\left(2\right)},...,t^{\left(|T|\right)}\}$ is a finite partition of T, then by Theorem 4 we can enclose range(*f*; ***t***^(*i*)^), i.e. the range of *f *over the *i*-th element of T, with the interval extension **f **of f:

(13) $$\text{range}(f;t^{\left(i\right)})\subseteq f(t^{\left(i\right)}):=[\underline{f}(t^{\left(i\right)}),\bar{f}(t^{\left(i\right)})],\forall i\in \{1,2,...,|T|\}.$$

For a given partition T, we can construct a partition-specific envelope function:

(14) $$\begin{array}{cc} {\hat{g}}^{T}(t)=\sum_{i=1}^{\left|T\right|}\bar{f}(t^{\left(i\right)})1_{\left\{t\in t^{\left(i\right)}\right\}}, & 1_{\left\{t\in t^{\left(i\right)}\right\}}=\{\begin{array}{ll} 1 & \text{if }t\in t^{\left(i\right)}\\ 0 & \text{otherwise}. \end{array} \end{array}$$

The necessary envelope condition (1) is satisfied by ${\hat{g}}^{T}$(*t*) because of (13). We can obtain the corresponding proposal $g^{T}$(*t*) as a normalized simple function over T:

(15) $$g^{T}(t)={\left(N_{{\hat{g}}^{T}}\right)}^{-1}{\hat{g}}^{T}(t)={\left(N_{{\hat{g}}^{T}}\right)}^{-1}\sum_{i=1}^{\left|T\right|}\bar{f}(t^{\left(i\right)})1_{\left\{t\in t^{\left(i\right)}\right\}},$$

where the normalizing constant $N_{{\hat{g}}^{T}}:=\sum_{i=1}^{\left|T\right|}\left(\text{vol }t^{\left(i\right)}\cdot \bar{f}(t^{\left(i\right)})\right)$ and $\text{vol }t:=\prod_{i=1}^{n}\text{wid }t_{i}$ is the *volume *of the box ***t***. The volume of an interval ***x ***is simply its width, i.e. vol ***x ***= wid ***x***, if ***x *∈ **Iℝ. Now, we have all the ingredients to perform a more efficient, partition-specific, auto-validating von Neumann rejection sampling or simply Moore rejection sampling.

Before making formal statements about our sampler let us gain geometric insight into the sampler from Example 3 and Figure 4. The upper boundaries of rectangles of a given color, depicting a simple function in Figure 4, is a partition-specific envelope function (14) for the logarithm of the posterior shape or the log-likelihood function of Example 3 over the prior-specified support [10^-10^, 10] ⊂  0T. In Figure 4 only a small interval about the maximum likelihood estimate (red dot) that contains the posterior samples (gray '+' markers) is depicted since the likelihood falls sharply outside this range. Normalization of the envelope gives the corresponding proposal function (15). As the refinement of the domain proceeds through adaptive bisections (described later), the partition size increases. We show partitions of size 3,7 and 19 over an interval containing the posterior samples. These samples were obtained from the partition with 19 intervals. Each of the corresponding envelope functions (upper boundaries of rectangles of a given color) can be used to draw independent and identically distributed samples from the target posterior density. Note how the acceptance probability (ratio of the area below the target shape to that below the envelope) increases with refinement.

Theorem 6 shows that Moore rejection sampler (MRS) indeed produces independent samples from the desired target and Theorem 7 describes the asymptotics of the acceptance probability as the partition of the domain is refined. Proofs for both Theorems are included in the Appendix for completeness.

**Theorem 6 ***Suppose that the DAG expression *f *of the target shape f has a well-defined natural interval extension ***f ***over *$T\in I{\mathbb{R}}^{n}$. *If T is generated according to Algorithm 1, and if the the envelope function *${\hat{g}}^{T}$(*t*) *and the proposal density *$g^{T}$(*t*) *are given by *(14) *and *(15), *respectively, then T is distributed according to the target density f*^• ^: T ↦ ℝ.

Next we bound the partition-specific acceptance probability $A(T):A({\hat{g}}^{T})$ for this sampler. For simplicity, let the domain T of the target shape *f *be an interval. Due to the linearity of the integral operator and (13),

$$\begin{array}{lll} N_{f} & := & \int_{T}f(t)dt\\ & = & \sum_{i=1}^{\left|T\right|}\int_{t^{\left(i\right)}}f(t)dt\\ & \in & \sum_{i=1}^{\left|T\right|}\left(\text{wid }(t^{\left(i\right)})\cdot f(t^{\left(i\right)})\right)\\ & = & [\sum_{i=1}^{\left|T\right|}(\text{wid }(t^{\left(i\right)})\cdot \underline{f}(t^{\left(i\right)})),\sum_{i=1}^{\left|T\right|}(\text{wid }(t^{\left(i\right)})\cdot \bar{f}(t^{\left(i\right)}))]. \end{array}$$

Therefore,

(16) $$A(T):=A({\hat{g}}^{T})=\frac{N_{f}}{N_{{\hat{g}}^{T}}}=\frac{N_{f}}{\sum_{i=1}^{\left|T\right|}\left(\text{wid }(t^{\left(i\right)})\cdot \bar{f}(t^{\left(i\right)})\right)}\geq \frac{\sum_{i=1}^{\left|T\right|}\left(\text{wid }(t^{\left(i\right)})\cdot \bar{f}(t^{\left(i\right)})\right)}{\sum_{i=1}^{\left|T\right|}\left(\text{wid }(t^{\left(i\right)})\cdot \bar{f}(t^{\left(i\right)})\right)}.$$

If *f *∈ $E_{L}$, the Lipschitz class of elementary functions (Definition 6), then we might expect the enclosure of *N*_*f *_to be proportional to the *mesh *$mesh\;w:=\max_{i\in \{1,...,T\}}\text{wid }(t^{\left(i\right)})$ of the partition T.

**Theorem 7 ***Let *$U_{W}$*be the uniform partition of *T = [$\underline{t}$, $\bar{t}$] *into W intervals each of width w*

$$\begin{array}{lll} w & = & \frac{\left(\bar{t}-\underline{t}\right)}{W}\\ t_{W}^{\left(i\right)} & = & [\underline{t}+(i-1)w,\underline{t}+iw],i=1,...,W\\ U_{W} & = & \{t_{W}^{\left(i\right)},i=1,...,W\}. \end{array}$$

*and let f *∈ $E_{L}$, *then*

$$A(U_{W})=1-O(1/W)$$

Theorem 7 shows that if *f *∈ $E_{L}$ and $U_{W}$ is a uniform partition of T into *W *intervals, then the acceptance probability $A(U_{W})=1-O(1/W)$. Thus, the acceptance probability approaches 1 at a rate that is no slower than linearly with the mesh.

#### Prioritized partitions and pre-processed proposals

We studied the efficiency of uniform partitions for their mathematical tractability. In practice, we may further increase the acceptance probability for a given partition size by adaptively partitioning T. In our context, adaptive means the possible exploitation of any current information about the target. We can refine the current partition $T_{\alpha }$ and obtain a finer partition $T_{\alpha^{\prime }}$ with an additional box by bisecting a box $t^{\left(*\right)}$ ∈ $T_{\alpha }$ along the midpoint of its side with the maximal width into a left box $t_{L}^{\left(*\right)}$ and a right box $t_{R}^{\left(*\right)}$. There are several ways to choose a box $t^{\left(*\right)}$ ∈ $T_{\alpha }$ for bisection. For instance, a relatively optimal choice is

(17) $$t^{\left(*\right)}=\underset{t^{\left(i\right)}\in T_{\alpha }}{\arg\max}\left(\text{vol }(t^{\left(i\right)})\cdot \text{wid }(f(t^{\left(i\right)})\right).$$

We employ a priority queue to conduct sequential refinements of T under this partitioning scheme. This approach avoids the exhaustive argmax computations to obtain the ***t***^(*) ^for bisection at each refinement step. Thus, the current partition is represented by a queue of boxes that are prioritized in descending order by the the priority function vol (***t***^(*i*)^) · wid (**f**(***t***^(*i*)^) in (17). Therefore, the box with the largest uncertainty in the enclosure of the integral over it gets bisected first. There are several ways to decide when to stop refining the partition. A simple strategy is to stop when the number of boxes reaches a number that is well within the memory constraints of the computer, say 10^6^, or when the lower bound of the acceptance probability given by (16) is above a desired threshold, say 0.1.

Once we have a partition T of T, we can sample *t *from the proposal density $g^{T}$ given by (15) in two steps:

1. Sample a box ***t***^(*i*) ^∈ T according to the discrete distribution:

(18) $$\begin{array}{cc} {\ddot{g}}^{T}(t^{\left(i\right)})=\frac{\text{vol }t^{\left(i\right)}\bar{f}(t^{\left(i\right)})}{\sum_{i=1}^{\left|T\right|}\left(\text{vol }t^{\left(i\right)}\bar{f}(t^{\left(i\right)})\right)}, & t^{\left(i\right)}\in T, \end{array}$$

2. Sample a point *t *uniformly at random from the box ***t***^(*i*)^.

Sampling from large discrete distributions (with million states or more) can be made faster by pre-processing the probabilities and saving the result in some convenient look-up table. This basic idea [28] allows samples to be drawn rapidly. We employ an efficient pre-processing strategy known as the Alias Method [4] that allows samples to be drawn in constant time even for very large discrete distributions as implemented in the GNU Scientific Library [29]. We also minimize the number of evaluations of the target shape *f *by saving the box-specific computations of $\underline{f}$(***t***^(*i*)^) and $\bar{f}$(***t***^(*i*)^) and exploiting the so-called "squeeze principle", i.e. immediately accepting those points proposed in the box ***t***^(*i*) ^that fall below $\underline{f}$(***t***^(*i*)^) when uniformly stretched toward $\bar{f}$(***t***^(*i*)^).

Thus, by means of priority queues and look-up tables we can efficiently manage our adaptive partitioning of the domain for envelope construction, and rapidly draw samples from the proposal distribution. Our sampler class MRSampler implemented in MRS 0.1.2, a C++ class library for statistical set processing, builds on C-XSC 2.0, a C++ class library for extended scientific computing using interval methods [30]. All computations were done on a 2.8 GHz Pentium IV machine with 1 GB RAM. Having given theoretical and practical considerations to our Moore rejection sampler, we are ready to draw samples from various targets over small tree spaces.

## Results

The natural interval extension of the likelihood function over labeled boxes in the tree space allows us to employ the Moore rejection sampler to rigorously draw independent and identically distributed samples from the posterior distribution over a compact box in the tree space given by our prior distribution. We draw samples from the posterior distribution based on two mitochondrial DNA data sets and use these samples (i) to estimate the posterior probabilities of each of the three rooted topologies, (ii) to conduct a nonparametric test of rate homogeneity between protein-coding and tRNA-coding sites and (iii) to estimate the human-neanderthal divergence time.

### Human, chimpanzee and gorilla

We revisit the data from a segment of the mitochondrial DNA of human, chimpanzee and gorilla [27] that was analyzed under the CFN model of DNA mutation (Model 1) within a point estimation setting [17]. The sufficient statistics of pattern counts for this data with total number of sites *v *= 895 under the CFN model over the space of all three-leaved phylogenetic trees are:

(*c*_xxx_, *c*_xxy_, *c*_yxx_, *c*_xyx_) = (762, 54, 41, 38)

Let human, chimpanzee and gorilla be denoted by leaf labels 1, 2 and 3, or H, C and G, respectively. Let the set of rooted tree labels corresponding to (ii),(iii) and (iv) of Figure 1 be K = {1, 2, 3}. The maximum likelihood estimate over TK:=T1∪T2∪T3, the rooted and clocked three-leaved phylogenetic tree space, is derived in [17] as

t1^:=( 1t0^, 1t1^)=arg⁡max⁡( it0, it1)∈TKf( it0, it1)=(0.010036,0.048559).

Recall that due to our flat priors, our posterior shape *f*(^*i*^*t*):= *f*(^*i*^*t*_0_, ^*i*^*t*_1_) with *i *∈ K = {1, 2, 3} is our likelihood function over TK. Now, suppose  b1t(1), b2t(2),..., bnt(n) are *n *independent and identically distributed samples from the posterior density *f*^•^. over TK. We can obtain asymptotically consistent estimates of the posterior probabilities of T1, T2 and T3 from Monte Carlo integration of the indicator function of each of the three topology labels using

Pj^n:=1n∑i=1n1{bi=j}(tbi)→PPj:=∫TK1{i=j}(ti)f·(ti)∂(ti),1{bi=j}(tbi (i))={1if bi=j0otherwise.

The 95% confidence interval for ^*j*^*P*, based on asymptotic normality of the Monte Carlo estimator, is

Pj^n±1.96Pj^n(1−Pj^n)/n.

Point estimate and a symmetric 95% confidence interval for the posterior probability of each of the three topologies from *n *= 10^6 ^posterior samples are

P1^106=0.8875±0.0006,P2^106=0.0646±0.0005,P3^106=0.0479±0.0004.

**Figure 5:**
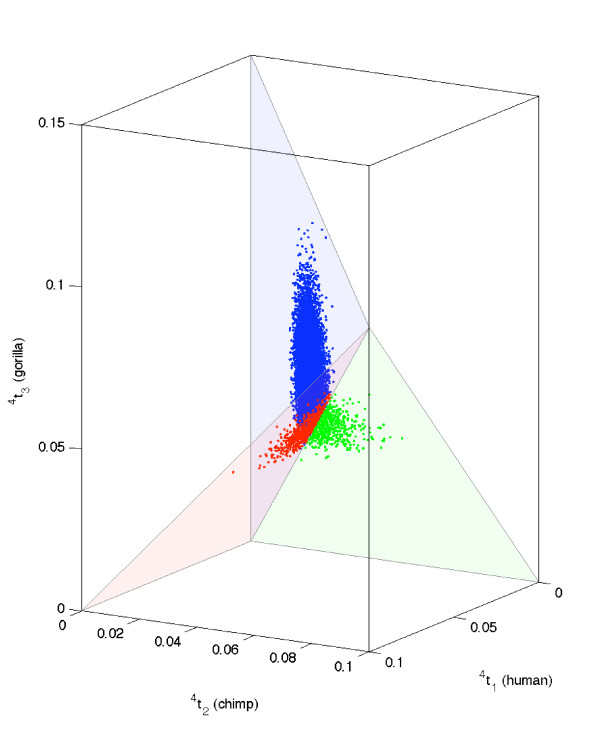
**Posterior samples from the rooted tree space of human, chimpanzee and gorilla**. Ten thousand independent and identically distributed posterior samples from the rooted and clocked binary tree space of human, chimpanzee and gorilla with topology label set {1 ∪ 2 ∪ 3} (see Figure 1(ii),(iii),(iv)) on the basis of mitochondrial data [27] summarized by (*c*_xxx_, *c*_xxy_, *c*_yxx_, *c*_xyx_) = (762, 54, 41, 38) under the Cavender-Farris-Neyman model (blue ∪ red ∪ green dots, respectively) are depicted.

These point estimates are in agreement with estimates obtained in [31,32] through quadrature routines in Mathematica. The first 10,000 of these samples are shown in Figure 5 upon transforming the rooted and clocked trees, ^*i*^*t *:= (^*i*^*t*_0_, ^*i*^*t*_1_), *i *∈ {1, 2, 3}, into constrained unrooted trees, ^4^*t *:= (^4^*t*_1_, ^4^*t*_2_, ^4^*t*_3_), according to Table 1.

**Table 1:** Rooted triplets as constrained unrooted triplets

Rooted and Clocked Trees, ^*i*^*t *:= (^*i*^*t*_0_, ^*i*^*t*_1_), *i *∈ {1, 2, 3}		Unrooted Trees ^4^*t *:= (^4^*t*_1_, ^4^*t*_2_, ^4^*t*_3_)		
Labeled Tree	Newick Representation of ^*i*^*t*	^4^*t*_1_	^4^*t*_2_	^4^*t*_3_

^1^*t *:= (^1^*t*_0_, ^1^*t*_1_)	((H:^1^*t*_1_, C:^1^*t*_1_):^1^*t*_0_, G:^1^*t*_0 _+ ^1^*t*_1_))	^1^*t*_1_	^1^*t*_1_	^1^*t*_1 _+ ^1^*t*_0 _+ ^1^*t*_0_

^2^*t *:= (^2^*t*_0_, ^2^*t*_1_)	((C:^2^*t*_1_, G:^2^*t*_1_):^2^*t*_0_, H:^2^*t*_0 _+ ^2^*t*_1_))	^2^*t*_1 _+ ^2^*t*_0 _+ ^2^*t*_0_	^2^*t*_1_	^2^*t*_1_

^3^*t *:= (^3^*t*_0_, ^3^*t*_1_)	((H:^3^*t*_1_, G:^3^*t*_1_):^3^*t*_0_, C:^3^*t*_0 _+ ^3^*t*_1_))	^3^*t*_1_	^3^*t*_1 _+ ^3^*t*_0 _+ ^3^*t*_0_	^3^*t*_1_

Any labeled, rooted and clocked tree with three leaves can be represented as a constrained unrooted tree according to the tabulated transformation.

Obtaining confidence intervals from dependent MCMC samples requires nontrivial computations for the burn-in period and the thinning rate [1]. These are not readily available for phylogenetic MCMC samplers. Thus, the independent and identically distributed samples from our rejection sampler has the advantage of producing valid confidence intervals for our integrals of interest. The point estimate of the posterior mean E(T1):=∫T1t1 f(t1)∂(t1) for topology label 1 is (0.010863, 0.048994). This posterior mean is close to (0.010036, 0.048559), the mode of our target shape or the maximum likelihood estimate derived in [17].

### Chimpanzee, gorilla and orangutan

We focus here on the 895 bp long homologous segment of mitochondrial DNA from chimpanzee, gorilla and orangutan [27]. This gives us a greater phylogenetic depth than the human, chimpanzee and gorilla sequences that were just analyzed. These sequences encode the genes for three transfer RNAs and parts of two proteins. Under the assumption of independence across sites, the sufficient statistics, under the JC model of DNA mutation (Model 2) over triplets, are given in Table 2 for all of the data as well as a partition of the data into tRNA-coding and protein-coding sites.

**Table 2:** Minimal sufficient statistics for the chimpanzee, gorilla and orangutan data

Site type	*v*	*c*_xxx_	*c*_xxy_	*c*_yxx_	*c*_xyx_	*c*_xyz_
All	895	700	100	46	42	7
tRNA-coding	198	173	13	7	3	2
protein-coding	697	527	87	39	39	5

The minimal sufficient statistics under the JC model for all 895 sites, 198 tRNA-coding sites and 697 protein-coding sites based on the homologous segment of mitochondrial DNA from chimpanzee, gorilla and orangutan [27].

**Figure 6:**
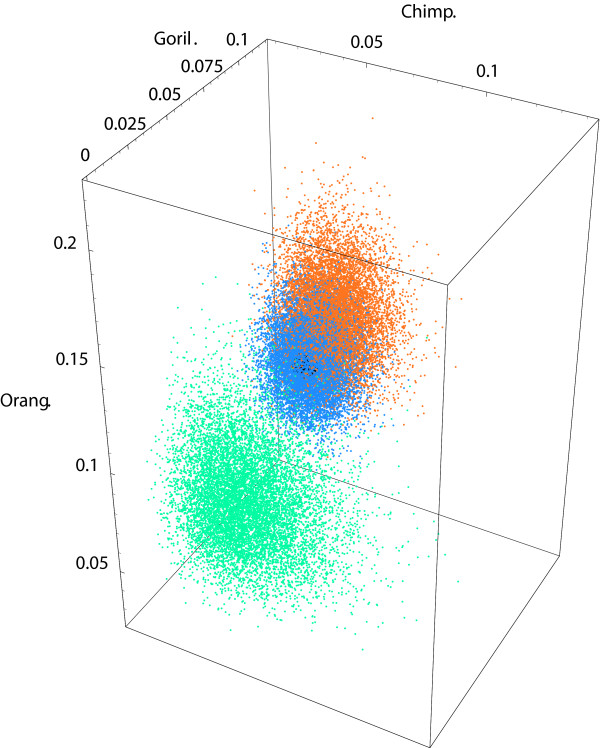
**Posterior samples from the unrooted tree space of chimpanzee, gorilla and orangutan**. Ten thousand Moore rejection samples from the posterior distribution over the three branch lengths of the unrooted tree space of chimpanzee, gorilla and orangutan based on their homologous mitochondrial DNA sequence of length 895 base pairs (blue dots), the tRNA-coding sequence with 198 base pairs (green dots) and the protein-coding sequence with 697 base pairs (red dots). The verified maximum likelihood estimate is the large black dot within the blue dots.

Ten thousand independent and identically distributed samples were drawn in 942 CPU seconds from the posterior distribution over JC triplets, i.e. unrooted trees with three edges corresponding to the three primates. Figure 6 shows these samples (blue dots) scattered about the verified global maximum likelihood estimate (MLE) of the triplet obtained in [5,26] and subsequently confirmed algebraically in [23]. We also drew ten thousand independent and identically distributed samples from the posterior based on the 198 tRNA-coding DNA sites (green dots in Figure 6) as well as from that based on the remaining 697 protein-coding sites (red dots in Figure 6). The former posterior samples, corresponding to the tRNA-coding sites, are more dispersed than the posterior samples based on the entire sequence. This is due to the smaller number of tRNA-coding sites making the posterior less concentrated. Moreover, the cluster of samples from the posterior based on tRNA-coding sites seem to be farther away from that based on protein-coding sites. Such a clustering of two sets of posterior samples is a signal of mutational rate heterogeneity between the two types of sites. Hotelling's trace statistics, being a natural measure of distance between two clusters of points, can be used as a test statistic to determine the significance of the observed test statistic. On the basis of 100 random permutations of the sites, we obtain the null distribution of Hotelling's trace statistics. We were able to reject the null hypothesis of rate homogeneity between the posterior samples based on the tRNA-coding sites and that based on the protein-coding sites at the 10% significance level using this permutation test (P-value = 0.06). Any biological interpretation of this test must be done cautiously since the JC model employed here forbids any transition:transversion bias that is reportedly relevant for this data [27].

### Neanderthal, human and chimpanzee

We used the 15 site patterns and their counts in Table 3 to infer the human-neanderthal divergence time. These counts are obtained from a multiple sequence alignment of the data made available in [33]. Our alignment procedure is more robust at the ends of each locus than that of [33]. We do an ordered concatenation of all the loci for each species prior to a multiple sequence alignment. The alignment was further edited by hand to obtain the locus-specific alignments. Under the assumption of independence across sites, the sufficient statistics, under any Markov model of DNA mutation, is the set of distinct site patterns and their respective counts. They are given in Table 3 for this data set.

**Table 3:** Minimal sufficient statistics for the neanderthal, human and chimpanzee data

Site	: 0 0 0 0 0 0 0 0 0 1 1 1 1 1 1
Pattern	: 1 2 3 4 5 6 7 8 9 0 1 2 3 4 5
. . . . . . . . . . . . . . . . . . . . .	
neanderthal	: a t c g a t c g t t g a c a a
human	: a t c g a t c g t c a g t a g
chimpanzee	: a t c g g c t a a t a a c t g
. . . . . . . . . . . . . . . . . . . . .	
site	: 6 6 6 4 1 1 1 1 2 2 1 1 1 1 1
pattern	: 8 0 0 5 5 4 4 0
counts	: 5 5 3 0

Site patterns and their counts from a multiple sequence alignment of the whole mitochondrial genome shotgun sequence (gi|115069275) of a neanderthal fossil Vi-80, from Vindija cave, Croatia [33], and its homologous sequence in a human (gi|13273200) and a chimpanzee (gi|1262390). The first column, (01.aaa.685)^*T*^, expresses that there are 685 sites with nucleotide a in all three species,..., and the fif-teenth column, (15.agg.1)^*T*^, expresses that there is 1 site with nucleotide g in human and chimpanzee and nucleotide a in neanderthal.

**Figure 7:**
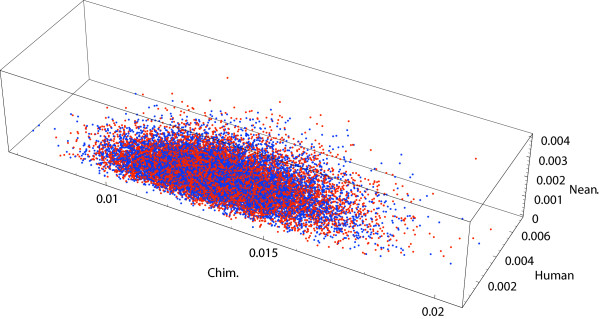
**Posterior samples from the unrooted tree space of neanderthal, human and chimpanzee**. Ten thousand Moore rejection samples each from the posterior distribution over the three branch lengths of the unrooted tree space of neanderthal, human and chimpanzee under the JC model (blue dots) and the HKY model (red dots).

We drew 10,000 samples that were independently and identically distributed from each of three posterior densities; (i) over the space of unrooted triplets under the JC model in 312 CPU seconds, (ii) over the clocked and rooted triplets under the JC model in 375 CPU seconds and (iii) over the clocked and rooted triplets under the HKY model in 1.2 CPU hours. In the HKY model we used the empirical nucleotide frequencies from the data (*π*(T) = 0.2588, *π*(C) = 0.2571, *π*(A) = 0.2916, *π*(G) = 0.1925) and a hominid-specific transition:transversion rate of 2.0. Unlike the JC model with five sufficient statistics (*c*_xxx_, *c*_xxy_, *c*_yxx_, *c*_xyx_, *c*_xyz_) = (2343, 56, 2, 4, 0), all 15 distinct site patterns are required for the likelihood computations under the HKY model and this is reflected in its longer CPU time. Both models gave similar posterior samples over rooted triplets, as shown in Figure 7.

We transformed the three posterior distributions over the triplet spaces; (i) unrooted JC triplets that were rooted using the mid-point rooting method, (ii) rooted JC triplets and (iii) rooted HKY triplets, respectively, into three posterior distributions over the human-neanderthal divergence time relative to the human-chimp divergence time. The corresponding posterior quantiles ({5%, 50%, 95%}) for the human-neanderthal divergence time in units of human-chimp divergence time are {0.0643, 0.125, 0.214}, {0.0694, 0.142, 0.263} and {0.0682, 0.143, 0.268}, respectively. We constrained the neanderthal lineage to be a fraction of the human lineage in branch length in order to estimate the age of the neanderthal fossil from the rooted HKY triplets. The posterior quantiles of the fossil date in units of human-chimp divergence is {0.00685, 0.0666, 0.195}. The estimate of 38; 310 years based on carbon-14 accelerator mass spectrometry [33] is within our [5%, 95%] posterior quantile interval for the fossil date, provided the human-chimp divergence estimate ranges in [196103, 5.6 × 10^6^]. Thus, reasonable bounds for the human-chimp divergence are 4 × 10^6 ^and 5.6 × 10^6 ^years, under the assumption that 4 × 10^6 ^is an acceptable lower-bound. Based on these two calendar year estimates, we transformed the posterior quantiles of the human-neanderthal divergence times from the rooted HKY triplets into {272680, 571124, 1073375} and {381752, 799574, 1502724} years, respectively. Our [5%, 95%] posterior intervals contain the interval estimate of [461000, 825000] years reported in [33]. However, our confidence intervals are from perfectly independent samples from the posterior and account for the finite number of neanderthal sites that were successfully sequenced, unlike those obtained on the basis of a bootstrap of site patterns [34] or heuristic MCMC [1]. Unfortunately, our human-neanderthal divergence estimates are overestimates as they ignore the non-negligible time to coalescence of the human and neanderthal homologs within the human-neanderthal ancestral population. Improvements to our estimates based on the other 310 human and 4 chimpanzee homologs reported in [33] may be possible with more sophisticated models of populations within a phylogeny and needs further investigation.

### Chimpanzee, gorilla, orangutan and gibbon

We were able to draw samples from JC quartets on the basis of the mitochondrial DNA of chimpanzee, gorilla, orangutan and gibbon [27]. The data for all four primates can be summarized by 61 distinct site patterns [5]. Now, the problem is more challenging because there are three distinct tree topologies in the unrooted, bifurcating, quartet tree space, and each of these topologies has five edges. Thus, the domain of quartets is a piecewise Euclidean space that arises from a fusion of 3 distinct five dimensional orthants. Since the post-order traversals (Algorithm 2) specifying the likelihood function are topology-specific, we extended the likelihood over a compact box of quartets in a topology-specific manner. The computational time was about a day and a half to draw 10000 samples from the quartet target due to low acceptance probability of the naive likelihood function based on the 61 distinct site patterns. All the samples had the topology which grouped Chimp and Gorilla together, i.e. ((chimpanzee, gorilla), (orangutan, gibbon)). The samples (results not shown) were again scattered about the verified global MLE of the quartet [5]. This quartet likelihood function has an elaborate DAG with numerous operations. When the data got compressed into sufficient statistics, the efficiency increased tremendously (e.g. for triplets the efficiency increases by a factor of 3.7). This is due to the number of leaf nodes in the target DAG, which encode the distinct site patterns of the observed data into the likelihood function, getting reduced from 29 to 5 for the triplet target and from 61 to 15 for the quartet target [24].

## Discussion

Interval methods provide for a rigorous sampling from posterior target densities over small phylogenetic tree spaces. When one substitutes conventional floating-point arithmetic for real arithmetic in a computer and uses discrete lattices to construct the envelope and/or proposal, it is generally not possible to guarantee the envelope property, and thereby ensure that samples are drawn from the desired target density, except in special cases [35]. Thus, the construction of the Moore rejection sampler through interval methods, that enclose the target shape over the entire real continuum in any box of the domain with machine-representable bounds, in a manner that rigorously accounts for all sources of numerical errors (see [36] for a discussion on error control), naturally guarantees that the Moore rejection samples are independent draws from the desired target. Moreover, the target is allowed to be multivariate and/or non-log-concave with possibly 'pathological' behavior, as long as it has a well-defined interval extension. The efficiency of MRS is not immune to the curse of dimensionality and target DAG complexity. When the DAG expression for the likelihood gets large, its natural interval extension can have terrible over-enclosures of the true range, which in turn forces the adaptive refinement of the domain to be extremely fine for efficient envelope construction. Thus, a naive application of interval methods to targets with large DAGs can be terribly inefficient. In such cases, sampler efficiency rather than rigor is the issue. Thus, one may fail to obtain samples in a reasonable time, rather than (as may happen with non-rigorous methods) produce samples from some unknown and undesired target.

There are several ways in which efficiency can be improved for such cases. First, the particular structure of the target DAG should be exploited to avoid any redundant computations. For example, sufficient statistics must be used to dissolve symmetries in the DAG. Second, we can further improve efficiency by limiting ourselves to differentiable targets in *C*^*n*^. Tighter enclosures of the range of *f*(***t***^(*i*)^) with **f**(***t***^(*i*)^) can come from the enclosures of Taylor expansions of *f *around the midpoint mid (***t***^(*i*)^) through interval-extended automatic differentiation (e.g. [36]) that can then yield tighter estimates of the integral enclosures [37]. Third, we can employ pre-processing to improve efficiency. For example, we can pre-enclose the range of a possibly rescaled *f *over a partition of the domain and then obtain the enclosure of **f **over some arbitrary ***t ***through a combination of hash access and hull operations on the pre-enclosures. Such a pre-enclosing technique reduces not only the overestimation of target shapes with large DAGs but also the computational cost incurred while performing interval operations with processors that are optimized for floating-point arithmetic. In the next version of the MRS library we plan to extend interval arithmetic beyond $I{\mathbb{R}}^{n}$ to a class of multi-dimensional data-structures related to regular sub-pavings (e.g. [38]) to improve the efficiency of our sampler. Fourth, various contractors can be used to improve the range enclosure in polynomial time (e.g. [38]). The most promising contractors employ interval constraint propagation. Finally, efficiency at the possible cost of rigor can also be gained (up to 30%) by foregoing directed rounding during envelope construction.

Poor sampler efficiency makes it currently impractical to sample from trees with five leaves and 15 topologies. However, one could use such triplets and quartets drawn from the posterior distribution to stochastically amalgamate and produce estimates of larger trees via fast amalgamating algorithms (e.g. [39,40]), which may then be used to combat the slow mixing in MCMC methods [2] by providing a good set of initial trees. A collection of large trees obtained through such stochastic amalgamations would account for the effect of finite sample sizes (sequence length) as well as the sensitivity of the amalgamating algorithm itself to variation in the input vector of small tree estimates. It would be interesting to investigate if such stochastic amalgamations can help improve mixing of MCMC algorithms on large tree spaces, albeit auto-validating rejection sampling via the natural interval extension of the likelihood function may not be practical for trees with more than four leaves.

## Conclusion

None of the currently available punctual samplers can rigorously produce independent and identically distributed samples from the posterior distribution over phylogenetic tree spaces, even for 3 or 4 taxa. We describe a new approach for rigorously drawing samples from a target posterior distribution over small phylogenetic tree spaces using the theory of *interval analysis*. Our Moore rejection sampler (MRS), being an auto-validating von Neumann rejection sampler (RS), can produce independent samples from any target shape *f *whose DAG expression f has a well-defined natural interval extension **f **over a compact domain T. MRS is said to be auto-validating because it automatically obtains a proposal *g *that is easy to simulate from, and an envelope $\hat{g}$ that is guaranteed to satisfy the envelope condition (1). MRS can circumvent the problems associated with (i) heuristic convergence diagnostics in MCMC samplers and (ii) pseudo-envelopes constructed via non-rigorous punctual methods in rejection samplers. When the target DAG is large, MRS becomes inefficient and may fail to produce the desired samples in a reasonable time, rather than (as may happen with non-rigorous methods) produce samples from some unknown and undesired target. MRS solves the open problem of rigorously drawing independent and identically distributed samples from the posterior distribution over small rooted and unrooted phylogenetic tree spaces (3 or 4 taxa) based on any multiply-aligned sequence data.

## Competing interests

The authors declare that they have no competing interests.

## Authors' contributions

RS developed the basic algorithm, analyzed the data and wrote the first draft. TY improved the object-oriented interface and refined the final implementation of the algorithm. Both authors edited the manuscript.

## Appendix

### Likelihoods for the CFN model on unrooted triplets

Recall that the probability that Y mutates to R, or vice versa, in time *t *is *a*(*t*):= (1 - *e*^-2*t*^)/2 and the stationary distribution *π*(R) = *π*(Y) = 1/2. Next we apply Algorithm 2 to compute the likelihood $l_{d_{\cdot ,q}}$ at a given site *q *which could be one of *l*_0_(^4^*t*), *l*_1_(^4^*t*),..., *l*_7_(^4^*t*).

(19) l0(t4)=π(R)PR,R( 4t1)PR,R( 4t2)PR,R( 4t3)+π(Y)PY,R( 4t1)PY,R( 4t2)PY,R( 4t3)=12((1−a( 4t1))(1−a( 4t2))(1−a( 4t3))+a( 4t1)a( 4t2)a( 4t3))=116((1+e−2( 4t1))(1+e−2( 4t2))(1+e−2( 4t3))+(1−e−2( 4t1))(1−e−2( 4t2))(1−e−2( 4t3)))=18(1+e−2( 4t1+ 4t2)+e−2( 4t2+ 4t3)+e−2( 4t1+ 4t3))

(20) l1(t4)=π(R)PR,Y( 4t1)PR,Y( 4t2)PR,Y( 4t3)+π(Y)PY,Y( 4t1)PY,Y( 4t2)PY,Y( 4t3)=12(a( 4t1)a( 4t2)a( 4t3)+(1−a( 4t1))(1−a( 4t2))(1−a( 4t3)))=l0(t4)

(21) l2(t4)=π(R)PR,R( 4t1)PR,R( 4t2)PR,Y( 4t3)+π(Y)PY,R( 4t1)PY,R( 4t2)PY,Y( 4t3)=12((1−a( 4t1))(1−a( 4t2))a( 4t3)+a( 4t1)a( 4t2)(1−a( 4t3)))=116((1+e−2( 4t1))(1+e−2( 4t2))(1−e−2( 4t3))+(1−e−2( 4t1))(1−e−2( 4t2))(1+e−2( 4t3)))=18(1+e−2( 4t1+ 4t2)−e−2( 4t2+ 4t3)−e−2( 4t1+ 4t3))

(22) l3(t4)=π(R)PR,Y( 4t1)PR,Y( 4t2)PR,R( 4t3)+π(Y)PY,Y( 4t1)PY,Y( 4t2)PY,R( 4t3)=12(a( 4t1)a( 4t2)(1−a( 4t3))+(1−a( 4t1))(1−a( 4t2))a( 4t3))=l2(t4)

(23) l4(t4)=π(R)PR,R( 4t1)PR,Y( 4t2)PR,Y( 4t3)+π(Y)PY,R( 4t1)PY,Y( 4t2)PY,Y( 4t3)=12((1−a( 4t1))a( 4t2)a( 4t3)+a( 4t1)(1−a( 4t2))(1−a( 4t3)))=116((1+e−2( 4t1))(1−e−2( 4t2))(1−e−2( 4t3))+(1−e−2( 4t1))(1+e−2( 4t2))(1+e−2( 4t3)))=18(1−e−2( 4t1+ 4t2)+e−2( 4t2+ 4t3)−e−2( 4t1+ 4t3))

(24) l5(t4)=π(R)PR,Y( 4t1)PR,R( 4t2)PR,R( 4t3)+π(Y)PY,Y( 4t1)PY,R( 4t2)PY,R( 4t3)=12(a( 4t1)(1−a( 4t2))(1−a( 4t3))+(1−a( 4t1))a( 4t2)a( 4t3))=l4(t4)

(25) l6(t4)=π(R)PR,R( 4t1)PR,Y( 4t2)PR,R( 4t3)+π(Y)PY,R( 4t1)PY,Y( 4t2)PY,R( 4t3)=12((1−a( 4t1))a( 4t2)(1−a( 4t3))+a( 4t1)(1−a( 4t2))a( 4t3))=116((1+e−2( 4t1))(1−e−2( 4t2))(1+e−2( 4t3))+(1−e−2( 4t1))(1+e−2( 4t2))(1−e−2( 4t3)))=18(1−e−2( 4t1+ 4t2)−e−2( 4t2+ 4t3)+e−2( 4t1+ 4t3))

(26) l7(t4)=π(R)PR,Y( 4t1)PR,R( 4t2)PR,Y( 4t3)+π(Y)PY,Y( 4t1)PY,R( 4t2)PY,Y( 4t3)=12(a( 4t1)(1−a( 4t2))a( 4t3)+(1−a( 4t1))a( 4t2)(1−a( 4t3)))=l6(t4)

### Proof of Theorem 1 (cf. [37])

Since any real arithmetic operation *x *⋆ *y*, where ⋆ ∈ {+, - ×,/} and *x*, *y *∈ ℝ, is a continuous function *x *⋆*y *:= ⋆(*x, y*): ℝ ⊗ ℝ ↦ ℝ, except when *y *= 0 under / operation. Since ***x ***and ***y ***are simply connected compact intervals, so is their Cartesian product ***x *⊗ *y***. On such a domain ***x ***⊗ ***y***, the continuity of ⋆(*x, y*) (except when ⋆ =/and 0 ∈ ***y***) ensures the attainment of a minimum, a maximum and all intermediate values. Therefore, with the exception of the case when ⋆ = / and 0 ∈ ***y***, the range ***x ***⋆ ***y ***has an interval form [min (*x *⋆*y*), max (*x *⋆*y*)], where the min and max are taken over all pairs (*x, y*) ∈ ***x ***⊗ ***y***. Fortunately, we do not have to evaluate *x *⋆*y *over every (*x*, *y*) ∈ ***x ***⊗ ***y ***to find the global min and global max of ⋆(*x, y*) over ***x ***⊗ ***y***, because the monotonicity of the ⋆(x, y*) in terms of *x *∈ ***x ***for any fixed y* ∈ ***y ***implies that the extremal values are attained on the boundary of ***x ***⊗ ***y***, i.e. the set {*x*, *y*, $\bar{x}$, and $\bar{y}$}. Thus the theorem can be verified by examining the finitely many boundary cases.

### Proof of Theorem 2

***x ***⋆ ***y ***= {*x *⋆*y *: *x *∈ ***x***, *y *∈ ***y***} ⊆ {*x *⋆*y *: *x *∈ ***x'***, *y *∈ ***y'***} = ***x' ***⋆ ***y'***.

### Proof of Theorem 3 (cf. [37])

Since **f**(***y***) is well-defined, we will not run into division by zero, and therefore (i) follows from the repeated invocation of Theorem 2. We can prove (ii) by contradiction. Suppose range(*f*; ***x***) ⊈ **f**(***x***). Then there exists *x *∈ ***x***, such that *f*(*x*) ∈ range(*f*; ***x***) but *f*(*x*) ∉ **f**(***x***). This in turn implies that *f*(*x*) = **f**([*x, x*]) ∉ **f**(***x***), which contradicts (i). Therefore, our supposition cannot be true and we have proved (ii) range(*f*; ***x***) ⊆ **f**(***x***).

### Proof of Theorem 4 (cf. [37])

Any elementary function *f *∈ E with expression f is defined by the recursion 9 on its sub-expressions f_*i *_where *i *∈ {1,..., *n*} according to its DAG. If *f*(*x*) = *p*(*x*)/*q*(*x*) is a rational function, then the theorem already holds by Theorem 3, and if *f *∈ S then the theorem holds because the range enclosure is exact for standard functions. Thus it suffices to show that if the theorem holds for f_1_, f_2 _∈ E, then the theorem also holds for f_1 _⋆ f_2_, where ⋆ ∈ {+, -,/, ×, ◦}. By ◦ we mean the composition operator. Since the proof is analogous for all five operators, we only focus on the ◦ operator. Since **f **is well-defined on its domain ***y***, neither the real-valued f nor any of its sub-expressions f_*i *_has singularities in its respective domain ***y***_*i *_induced by ***y***. In particular f_2 _is continuous on any ***x***_2 _and ${x^{\prime }}_{2}$ such that ***x***_2 _⊆ ${x^{\prime }}_{2}$ ⊆ ***y***_2 _implying the compactness of **f**_2_(***x***_2_) =: ***w***_2 _and **f**_2_(${x^{\prime }}_{2}$) =: ${w^{\prime }}_{2}$, respectively. By our assumption that **f**_1 _and **f**_2 _are inclusion isotonic we have that ***w***_2 _⊆ ${w^{\prime }}_{2}$ and also that

$$f_{1}\circ f_{2}(x_{2})=f_{1}(f_{2}(x_{2}))=f_{1}(w_{2})\subseteq f_{1}({w^{\prime }}_{2})=f_{1}(f_{2}({x^{\prime }}_{2}))=f_{1}\circ f_{2}({x^{\prime }}_{2})$$

The range enclosure is a consequence of inclusion isotony by an argument identical to that given in the proof for Theorem 3.

### Proof of Theorem 5 (cf. [37])

The proof is given by an induction on the DAG for *f *similar to the proof of Theorem 4 (See [37]).

### Proof of Theorem 6

Let the domain T of the target *f*^• ^be an element of $I{\mathbb{R}}^{n}$. From (15) and (14) observe that ${\hat{g}}^{T}(t)=g^{T}(t)N_{{\hat{g}}^{T}}$. Let us define the following two subsets of ℝ^*n*+1^,

$$\begin{array}{ccc} ℬ({\hat{g}}^{T})=\{(v,u):v\in T,0\leq u\leq {\hat{g}}^{T}(v)\}, & \text{and} & ℬ(f)=\{(v,u):v\in T,0\leq u\leq f(v)\}. \end{array}$$

Algorithm 1 first produces a sample from the random vector (*V*, *U*) that is uniformly distributed in $ℬ({\hat{g}}^{T})$. We can see this by letting *h*(*v*, *u*) denote the joint density of (*V*, *U*) and *h*(*u|v*) denote the conditional density of *U *given *V *= *v*. Then,

$$h(v,u)=\{\begin{array}{ll} g^{T}(v)h(u|v) & \text{if }(v,u)\in ℬ({\hat{g}}^{T})\\ 0 & \text{otherwise}\text{.} \end{array}$$

Since we sample a height *u *for a given *v *from the Uniform [0, ${\hat{g}}^{T}$] distribution,

$$h(u|v)=\{\begin{array}{ll} {\left({\hat{g}}^{T}(v)\right)}^{-1}=(g^{T}(v))N_{{\hat{g}}^{T}}){-}^{1} & \text{if }u\in [0,{\hat{g}}^{T}(v)]\\ 0 & \text{otherwise}\text{.} \end{array}$$

Therefore,

$$h(v,u)=\{\begin{array}{ll} g^{T}(v)h(u|v)=g^{T}(v){\left(g^{T}(v)N_{{\hat{g}}^{T}}\right)}^{-1}={\left(N_{{\hat{g}}^{T}}\right)}^{-1} & \text{if }(v,u)\in ℬ({\hat{g}}^{T})\\ 0 & \text{otherwise}\text{.} \end{array}$$

Thus, we have shown that the joint density of the random vector (*V*, *U*) initially produced by Algorithm 1 is uniformly distributed on $ℬ({\hat{g}}^{T})$. The above relationship also makes geometric sense since the volume of $ℬ({\hat{g}}^{T})$ is exactly $N_{{\hat{g}}^{T}}$.

Now, let (*T*, *S*) be the accepted random vector during the accept/reject step of Algorithm 1, i.e.

$$(T,S)=(V,U)\Leftrightarrow (V,U)\in ℬ(f)\subseteq ℬ({\hat{g}}^{T}).$$

Then, the uniform distribution of (*V*, *U*) on $ℬ({\hat{g}}^{T})$ implies the uniform distribution of (*T*, *S*) on ℬ(*f*). Since the volume of ℬ(*f*) is *N*_*f*_, the density of (*T*, *S*) is identically 1/*N*_*f *_on ℬ(*f*) and 0 elsewhere. Hence, the marginal density of *T *on T is

$$\begin{array}{lll} \int_{0}^{f(t)}1/N_{f}\;dh & = & 1/N_{f}\int_{0}^{f(t)}1\;dh\\ & = & \begin{array}{cc} 1/N_{f}\int_{0}^{N_{f}f^{\cdot }(t)}1\;dh, & ∵f^{\cdot }(t)=f(t)/N_{f} \end{array}\\ & = & f^{\cdot }(t). \end{array}$$

Thus, we have shown that the accepted random vector *T *has the desired density *f*^•^.

### Proof of Theorem 7

Due to Theorem 5,

$$\begin{array}{lll} \text{wid }(t_{W}^{\left(i\right)})=O(1/W) & \Rightarrow & {\text{dist}}_{\infty }(\text{range }(f;t_{W}^{\left(i\right)},f(t_{W}^{\left(i\right)}))=O(1/W)\\ & \Rightarrow & \begin{array}{cc} \text{wid }(f(t_{W}^{\left(i\right)}))=O(1/W), & ∵f\in E_{ℒ} \end{array}. \end{array}$$

Therefore

$$\sum_{i=1}^{\left|U_{W}\right|}\left(\text{wid }(t_{W}^{\left(i\right)})\cdot f(t_{W}^{\left(i\right)})\right)=w\sum_{i=1}^{W}f([\underline{t}+(i-1)w,\underline{t}+iw]),$$

and we have

$$\begin{array}{ccc} \text{wid }(w\sum_{i=1}^{W}f(t_{W}^{i}))=O(1/W) & \Rightarrow & A(U_{W})=1-O(1/W). \end{array}$$

Therefore the lower bound for the acceptance probability ***A***($U_{W}$) of MRS approaches 1 no slower than linearly with the refinement of T by $U_{W}$. Note that this should hold for a general nonuniform partition with *w *replaced by the mesh.
